# Brain Exposure to Piperacillin in Acute Hemorrhagic Stroke Patients Assessed by Cerebral Microdialysis and Population Pharmacokinetics

**DOI:** 10.1007/s12028-020-00947-x

**Published:** 2020-03-26

**Authors:** Sami Ullah, Ronny Beer, Uwe Fuhr, Max Taubert, Markus Zeitlinger, Alexander Kratzer, Christoph Dorn, Usman Arshad, Mario Kofler, Raimund Helbok

**Affiliations:** 1 Department I of Pharmacology, Center for Pharmacology, Faculty of Medicine and University Hospital Cologne, University of Cologne, Cologne, Germany; 2 Neurological Intensive Care Unit, Department of Neurology, Medical University of Innsbruck, Innsbruck, Austria; 3 Department of Clinical Pharmacology, Medical University of Vienna, Waehringer Guertel 18-20, 1090 Vienna, Austria; 4 Hospital Pharmacy, University Hospital Regensburg, Regensburg, Germany; 5 Institute of Pharmacy, University of Regensburg, Regensburg, Germany; 6 Institute of Pharmacy, Clinical Pharmacy, University of Bonn, Bonn, Germany

**Keywords:** Piperacillin, Cerebral microdialysis, Acute hemorrhagic stroke, Population pharmacokinetics, Probability of target attainment

## Abstract

**Background:**

The broad antibacterial spectrum of piperacillin/tazobactam makes the combination suitable for the treatment of nosocomial bacterial central nervous system (CNS) infections. As limited data are available regarding piperacillin CNS exposure in patients without or with low-grade inflammation, a clinical study was conducted (1) to quantify CNS exposure of piperacillin by cerebral microdialysis and (2) to evaluate different dosing regimens in order to improve probability of target attainment (PTA) in brain.

**Methods:**

Ten acute hemorrhagic stroke patients (subarachnoid hemorrhage, *n* = 6; intracerebral hemorrhage, *n* = 4) undergoing multimodality neuromonitoring received 4 g piperacillin/0.5 g tazobactam every 8 h by 30-min infusions for the management of healthcare-associated pneumonia. Cerebral microdialysis was performed as part of the clinical neuromonitoring routine, and brain interstitial fluid samples were retrospectively analyzed for piperacillin concentrations after the first and after multiple doses for at least 5 days and quantified by high-performance liquid chromatography. Population pharmacokinetic modeling and Monte Carlo simulations with various doses and types of infusions were performed to predict exposure. A *T*_>MIC_ of 50% was selected as pharmacokinetic/pharmacodynamic target parameter.

**Results:**

Median peak concentrations of unbound piperacillin in brain interstitial space fluid were 1.16 (range 0.08–3.59) and 2.78 (range 0.47–7.53) mg/L after the first dose and multiple doses, respectively. A one-compartment model with a transit compartment and a lag time (for the first dose) between systemic and brain exposure was appropriate to describe the brain concentrations. Bootstrap median estimates of the parameters were: transfer rate from plasma to brain (0.32 h^−1^), transfer rate from brain to plasma (7.31 h^−1^), and lag time [2.70 h (coefficient of variation 19.7%)]. The simulations suggested that PTA would exceed 90% for minimum inhibitory concentrations (MICs) up to 0.5 mg/L and 1 mg/L at a dose of 12–16 and 24 g/day, respectively, regardless of type of infusion. For higher MICs, PTA dropped significantly.

**Conclusion:**

Limited CNS exposure of piperacillin might be an obstacle in treating patients without general meningeal inflammation except for infections with highly susceptible pathogens. Brain exposure of piperacillin did not improve significantly with a prolongation of infusions.

**Electronic supplementary material:**

The online version of this article (10.1007/s12028-020-00947-x) contains supplementary material, which is available to authorized users.

## Introduction

The broad antibacterial spectrum of the piperacillin/tazobactam combination would possibly qualify the combination for the treatment of nosocomial bacterial central nervous system (CNS) infections [1]. Achievement of concentrations suitable for treatment has been reported in cerebrospinal fluid (CSF) of meningitis patients [2] at a daily dose of 324–436 mg/kg body weight. However, patients without generalized meningeal inflammation, e.g., those with hydrocephalus, achieved insufficient concentrations in CSF [1]. Though CSF concentrations may sometimes reflect brain target site concentrations well, drug distribution might be different between CSF and extracellular fluid (ECF) of the brain [3] and may differ manifold between lumbar, ventricular, and cisternal parts of the compartment [4, 5]. Conversely, ECF concentrations measured through microdialysis reflect target site concentrations well as it only measures free (thus biologically active) concentrations. In the last two decades, the use of microdialysis has evolved quickly and is now an integral part of individualized intensive care therapy of acute injury patients undergoing multimodal monitoring in several centers [6]. Limited data are available regarding CNS exposure and related target attainment of antibiotics in patients with acute brain injuries.

Like other beta-lactam antibiotics, piperacillin exhibits time-dependent bacterial killing, with *T*_>MIC_ being the relevant pharmacokinetic/pharmacodynamic parameter [7]. Cefepime has been reported to show better exposure in terms of *T*_>MIC_ in plasma as well as in CSF when administered as a continuous infusion in contrast to intermittent infusion in neurosurgical patients with postoperative intracranial infections [8]. Piperacillin has also been reported to achieve better plasma exposure with continuous infusion or extended infusion as compared to intermittent infusion [9–12]. However, it is not yet known whether prolonged infusion can improve CNS exposure of piperacillin in brain.

Therefore, the objectives of this study were to quantify brain ECF concentrations of piperacillin using cerebral microdialysis and to develop a model to describe the kinetics of its brain exposure in patients without general meningeal inflammation. Subsequently, this model was used to predict the probability of target attainment (PTA) of different doses, types of infusions, and minimum inhibitory concentration (MIC) values in order to assess the pharmacokinetic fundament for a potential use of piperacillin in such patients.

## Methods

### Patient Selection and Ethical Approval

Ten comatose patients (median [range] age and body weight of 52 [32–72] years and 73 [60–95] kg) with acute hemorrhagic stroke (subarachnoid hemorrhage [SAH], *n* = 6; intracerebral hemorrhage [ICH], *n* = 4) admitted to the neurological intensive care unit (ICU) of the Department of Neurology at the Medical University of Innsbruck, Austria, requiring invasive multimodal neuromonitoring were recruited between October 2010 and November 2014 (Table 1). Patients were eligible if they developed healthcare-associated pneumonia and if antibacterial treatment with piperacillin/tazobactam was indicated. Sample size calculation was not performed prior to the study. The conduct of this study was approved by the Ethics Committee of the Medical University of Innsbruck (Approval Numbers AN3898 285/4.8, AM4091-292/4.6, UN3898 285/4.8) and registered with the institutional Clinical Trial Center (https://ctc.tirol-kliniken.at; study identifier 20131218-868). Additionally, the public at the research site was informed about the study by notice on the bulletin board at the neurological ICU. All provisions of the WMA Declaration of Helsinki in its applicable version were followed, and informed consent was obtained from all patients or legal representatives according to federal regulations. Clinical care of SAH and ICH patients strictly adhered to current international guidelines [13–15] with the exception of nimodipine being administered intravenously in SAH patients.

**Table 1:** Demographic and clinical characteristics of the patients

Parameter	Our study	Published plasma model used to develop the brain PK model [10]	
	Bolus infusion	Bolus infusion	Continuous infusion
Gender (male/female) (*n*)	2/8	5/3	6/2
Weight (kg)	73 (57–88)	80 (74–86)	73 (64–83)
Age (years)	52 (32–71)	41 (22–65)	30 (23–40)
Height (cm)	171 (166–176)	174 (172–180)	176 (171–177)
Piperacillin dose (mg day/kg)	166	229 (204–254)	168 (160–188)
Creatinine clearance (Cockcroft–Gault) (mL/min)	101.5 (63.5–139.5)	88.3(53.3–101.0)	96.7 (31.7–148.3)
APACHE II score on day 1	26 (24–31)	24 (18–26)	20 (16–22)
Stay in intensive care unit (days)	34 (18–50)	–	–

All values are given as median (inter-quartile range) except gender

*APACHE* Acute physiology and chronic health evaluation

### Data Collection, Neuromonitoring, and Sampling Procedures

Patient characteristics, medical complications, and outcome were prospectively recorded in the respective institutional SAH and ICH databases. In line with international consensus, patients underwent intracranial neuromonitoring including measurement of intracranial pressure, brain tissue oxygen tension, and cerebral metabolites based on clinical and radiological criteria [16]. A cerebral microdialysis catheter (71 High Cut-Off Brain Microdialysis Catheter, M Dialysis AB, Stockholm, Sweden) was tunneled and inserted into the white matter either “perilesionally” (i.e., placement of the catheter gold tip within 1 cm to a focal brain lesion) or otherwise into “normal-appearing tissue.” Isotonic perfusion fluid (Perfusion Fluid CNS; M Dialysis AB) was pumped through the microdialysis system at a flow rate of 0.3 µL/min. Hourly samples were analyzed with ISCUS^flex^ Point-of-Care Analyzer (M Dialysis AB) for interstitial glucose, pyruvate, lactate, and glutamate concentrations and frozen thereafter at − 80 °C. During the neuromonitoring period, all patients were intubated and mechanically ventilated. To facilitate mechanical ventilation, patients received continuous infusions of midazolam plus sufentanil and/or S-ketamine.

Immediately after diagnosis of healthcare-associated pneumonia, treatment was initiated according to local clinical infectious diseases guidelines with a 30-min intravenous infusion of 4 g piperacillin/0.5 g tazobactam (Fresenius Kabi, Graz, Austria) every 8 h. As stated, microdialysis samples of brain interstitial fluid were obtained in 1 h intervals both after first dose and after multiple doses at steady state (median of seven samples each). Importantly, cerebral microdialysis was performed as part of the clinical neuromonitoring routine and the cerebral microdialysis catheter remained in situ for the total neuromonitoring period, usually exceeding the span of piperacillin/tazobactam administration.

### Determination of In Vitro Recovery

Determination of recovery in vitro was performed using identical probes, flow rate, and perfusion fluid as in patients. In forward *µD* experiments, the microdialysis catheter was placed into the immersion solution containing piperacillin/tazobactam (1/0.125 [C1], 10/1.25 [C2], or 100/12.5 mg/L [C3]) and was constantly perfused with Perfusion Fluid CNS at a flow rate of 0.3 µL/min. In reverse *µD* experiments, immersion solutions contained plain Perfusion Fluid CNS, whereas the perfusion solution contained piperacillin/tazobactam. In both forward and reverse *µD* experiments, *µD* samples were collected over three consecutive sampling intervals of 60 min for two different probes.

### Analytical Assay

Due to the low flow rate (0.3 µL/min) used during clinical microdialysis, a sample volume of 18 µL for each hourly sample was obtained out of which approximately 6 µL was required for diagnostic purposes. The remaining sample volume was not sufficient for two analyses, and thus, tazobactam could not be quantified.

Piperacillin concentrations were determined by an isocratic high-performance liquid chromatography (HPLC) method with ultraviolet detection at 225 nm which has been validated according to the U.S. Food and Drug Administration (FDA) [17] and European Medicines Agency (EMA) [18] recommendations. Quality control samples of appropriate concentrations prepared in Ringer’s solution were analyzed with each assay. The coefficient of variation in intra- and inter-assay precision and accuracy was < 3% based on in-process quality controls (80, 8, and 0.8 mg/L). For liquid chromatography, an XBridge C18 BEH 2.5µ, 50 × 3 mm column with a Nucleoshell RP18 2.7µ, 4 × 3 mm guard column was used as the stationary phase. Isocratic elution was carried out with 0.1 M H_3_PO_4_, pH 2.7/acetonitrile 75:25 (v/v) using a flow rate of 0.4 mL/min. The lower limit of quantification (LLOQ) was 0.05 mg/L. Undiluted cerebral microdialysis samples were injected directly into the HPLC system, with a defined injection volume of 1–3 µL selected according to the expected concentration.

### Population Pharmacokinetic Analysis

The NONMEM software (version 7.4.3) [19] was used to develop a population pharmacokinetic (PopPK) model of piperacillin. Estimations were performed using first-order conditional estimation with interaction. Different models were assessed based on improvement in objective function value (drop of ≥ 3.84 corresponding to *p* < 0.05 with one degree of freedom, assuming a Chi-squared distribution), goodness-of-fit plots, and precision of parameter estimates from nonparametric bootstrap analysis of 1000 samples [20]. Visual predictive checks [21] were performed to assess the predictive performance of the models. Different models with one, two, and three compartments with or without lag time (*T*_lag_) and various number of transit compartments were tested to fit the brain ECF data.

Because plasma piperacillin concentrations were not available, a plasma model with similar study design and demographics was selected from the literature [10] to drive the brain concentrations in our model. Fixed effects parameters as well as inter-individual and intra-individual variability parameters were fixed in the model.

Dead space of the catheter (distance between semipermeable membrane of the probe and catheter outlet) was 5.1 µL, and thus, it would take approximately 0.28 h for the fluid to reach the outlet based on the flow rate (0.3 µL/min) used in the study. To account for the dead space, this time was subtracted from the end time of the microdialysis time (1-h interval) beforehand in the dataset. Microdialysis recovery was assumed to be 100% in the model based on the results of in vitro experiments. Piperacillin concentrations below LLOQ were retained in the dataset and evaluated with M3 and M5 methods [22].

The integrated approach [23] (numerical integration to calculate average concentrations during the corresponding microdialysis intervals) was used to model the brain data. Plasma protein binding was considered to be linear and was fixed to 30% according to the plasma pharmacokinetic model [10]. Inter-individual variability was estimated while assuming a log-normal distribution of parameters. Additive, proportional, and combined error models of residual unexplained variability (RUV) were evaluated on the model.

### Monte Carlo Simulations and Probability of Target Attainment (PTA)

Monte Carlo Simulations (MCS) (5000 simulated subjects) were performed based on the final PopPK model of piperacillin at various MIC levels (up to 16 mg/L), for three doses (12, 16, and 24 g/day) and three types of infusion (intermittent infusion over 30 min, extended infusion over 3 or 4 h, and continuous infusion). Based on plasma data, a pharmacokinetic/pharmacodynamic (PK/PD) index of *fT*_>MIC_ of 50% is considered to be essential for the optimal activity of piperacillin [24]. Therefore, *fT*_>MIC_ of 50% was selected as PK/PD index for the assessment of probability of target attainment against various pathogens according to the MIC distribution suggested by the European Committee on Antimicrobial Susceptibility Testing (EUCAST) for susceptible pathogens [25]. Minimum inhibitory concentration required to inhibit the growth of 50% (MIC_50_) and 90% (MIC_90_) of bacteria commonly involved in CNS infections was derived from MIC distribution of wild-type microorganisms on EUCAST Web site (Table 2) [25].

**Table 2:** MIC_50_ and MIC_90_ of piperacillin/tazobactam in mg/L against pathogens commonly involved in brain infections

Pathogen	MIC_50_^a^	MIC_90_^b^
*Haemophilus influenzae*	0.016	0.125
*Streptococcus agalactiae*	0.5	0.5
*Streptococcus pneumoniae*	0.016	0.5
*Neisseria meningitidis*	0.5	0.5
*Listeria monocytogenes*	2	4
*Staphylococcus aureus*	1	4
*Escherichia coli*	2	8
*Staphylococcus epidermidis*	2	16
*Pseudomonas aeruginosa*	8	128

MIC_50_^a^ and MIC_90_^b^ mean minimum inhibitory concentration required to inhibit the growth of 50% and 90% of organisms, respectively. These values have been derived from the wild-type distributions of bacteria listed on the EUCAST Web site (European Committee on Antimicrobial Susceptibility Testing. Data from the EUCAST MIC distribution Web site; Available from: https://mic.eucast.org/Eucast2/SearchController/search.jsp?action=performSearch&BeginIndex=0&Micdif=mic&NumberIndex=50&Antib=251&Specium=-1)

### Sensitivity Analysis

An additional evaluation (sensitivity analysis) was performed to avoid that the selected plasma model would bias simulated PTA in brain. To this end, the plasma model used was replaced by two other published plasma models [26, 27]. New parameters for the brain data were estimated, and MCS were done to assess PTA in brain as described above. These PTAs were then compared with those predicted in the previous simulations.

## Results

A total of 130 piperacillin brain concentrations from ten acute hemorrhagic stroke patients were available for the development of the PopPK model (median 0.95 mg/L [range 0.0–7.53]). Median peak concentrations of unbound piperacillin in brain interstitial space fluid after the first dose were 1.16 (range 0.08–3.59) mg/L. After multiple doses, median peak concentrations at steady state increased to 2.78 (range 0.47–7.53) mg/L. Mean recovery of piperacillin was 98.3 ± 12.9% (mean/SD).

### Brain ECF Pharmacokinetic Model

**Fig. 1:**
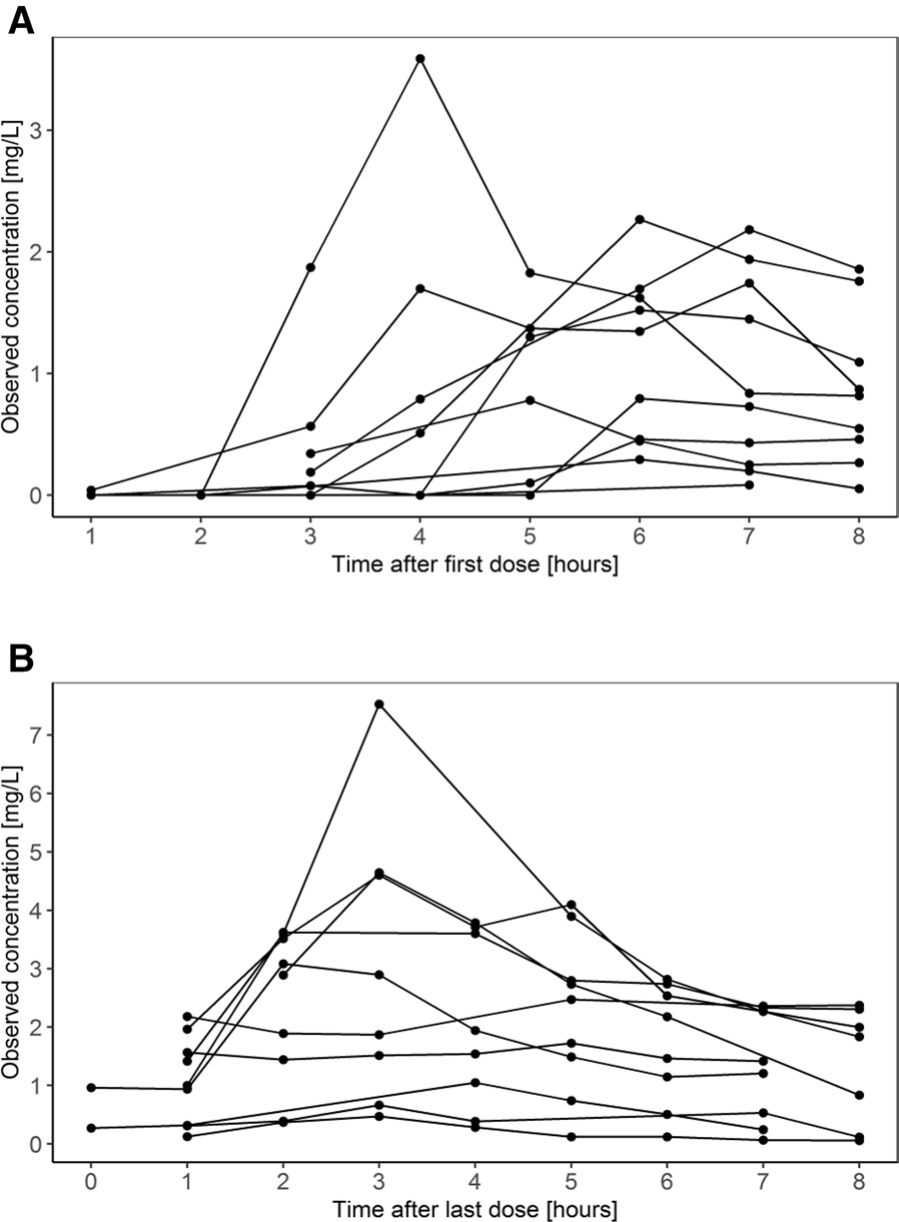
Individual piperacillin brain concentration versus time profiles showing high variability both after first dose (**a**) and after multiple doses (**b**)

**Fig. 2:**
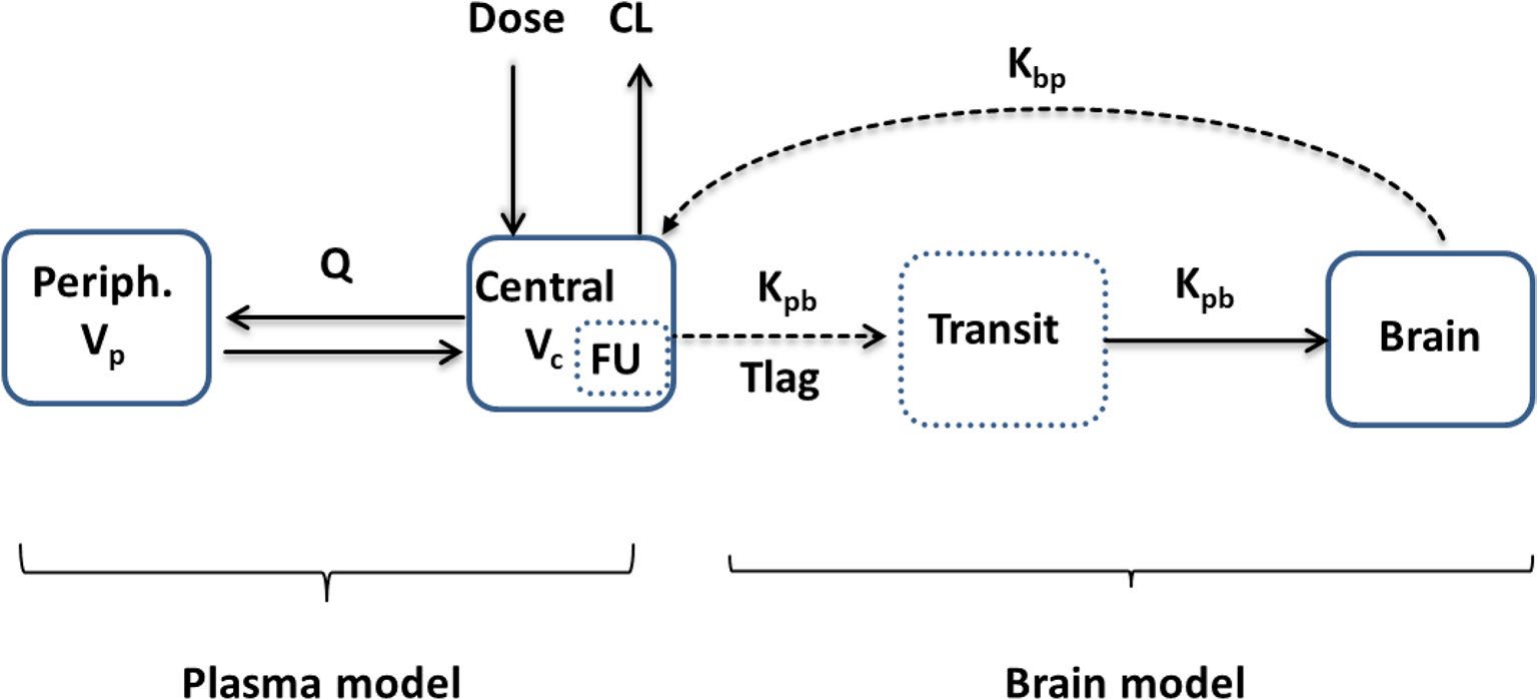
Model structure, where CL, *Q*, *V*_c_, and *V*_p_ are elimination clearance, inter-compartmental clearance, volume of distribution of central, and peripheral compartment of the plasma model, respectively. The unbound fraction (FU, fixed to 70%) of piperacillin in plasma was used to drive the brain concentrations. In the brain model, *K*_pb_, *K*_bp_, and *T*_lag_ represent transfer rate from plasma to brain, transfer rate from brain to plasma, and lag time, respectively. Continuous line denotes mass transfer between compartments

Brain concentrations of piperacillin were best described by a one-compartment model with first-order elimination. Based on the observed delay in the initial rise of brain concentrations (Fig. 1), a lag time (*T*_lag_) and a transit compartment between plasma and brain were added (Fig. 2). Neither *T*_lag_ nor the transit compartment alone was sufficient to describe the data. Multiple transit compartments did not improve the model further. Separate population estimates were generated for transfer rate from brain to plasma (*K*_bp_) and *T*_lag_ after the first dose and at steady state. At steady state, *T*_lag_ was not significantly different from zero; therefore, it was fixed to zero. A combined error model best described RUV in the model. Median estimates of the parameters with respective 95% confidence intervals (CI) are given in Table 3 from bootstrap analysis. In case of below quantification limit (BQL) concentrations, the M5 method performed similarly to the gold standard M3 method in terms of similar parameter estimates and goodness of fits. M5 was preferred for the final model because of numerical difficulties related to the M3 method.

**Table 3:** Pharmacokinetic parameters of the brain model of piperacillin

Parameter	Bootstrap estimates (samples = 1000) (median [95% CI])
Fixed effects	
*K*_pb_ (h^−1^)	0.32 (0.16–0.39)
*K*_bp_fd_ (h^−1^)	7.31 (3.47–12.9)
*K*_bp_md_ (h^−1^)	4.39 (1.35–10.6)
*T*_lag_fd_ (h)	2.70 (2.21–3.52)
*T*_lag_md_ (h)	0 FIXED
Inter-individual variability (CV %)	
*T*_lag_	19.7 (5.0–33.6)
Residual variability (SD)	
Additive error	0.03 (0.02–0.03)
Proportional error	0.35 (0.23–0.44)

*K*_pb_, *K*_bp_fd_, and *K*_bp_md_ represent rate of transfer from plasma to brain, transfer rate from brain to plasma after first dose, and transfer rate from brain to plasma after multiple doses, respectively. *T*_lag_fd_ and *T*_lag_md_ represent lag time after first and multiple doses, respectively

*CI* confidence interval, *CV* coefficient of variation, *SD* standard deviation

### Model Evaluation

**Fig. 3:**
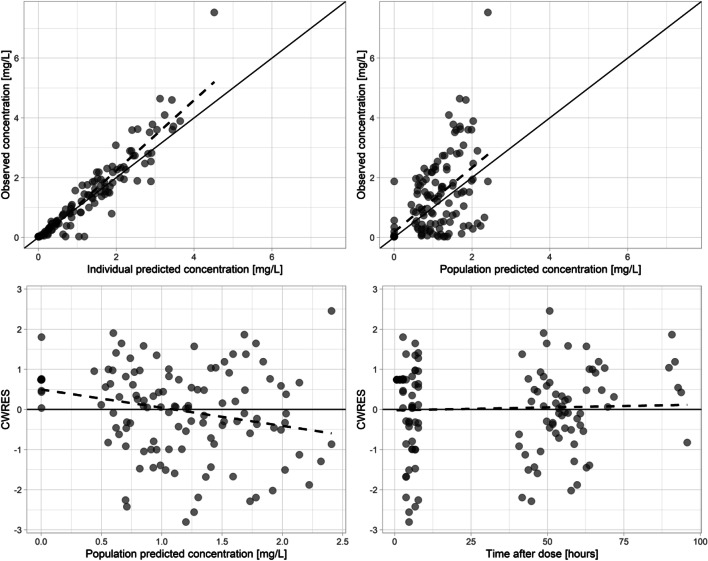
Combined goodness-of-fit plots of the piperacillin brain concentrations. Individual predictions versus observed concentrations (upper left), population predictions vs. observed concentration (upper right), population predictions versus conditional weighted residuals (lower left), and time after dose versus conditional weighted residuals (lower right)

Goodness-of-fit plots (Fig. 3) showed reasonable agreement between brain microdialysate observations and individual as well as population predictions for the final PopPK model. Conditional weighted residuals were uniformly distributed around zero; however, slight under-prediction at lower concentrations was visible. The M3 method did not improve the observed under-prediction at low concentrations either. Nonetheless, the model adequately described the central tendency and dispersion of the observed data appropriately (Fig S1 in supplementary materials). Concentration–time plots of individual patients in brain also indicated that model had appropriately described the data (Fig S2). Bootstrap results (Table 3) indicated that PK parameter estimates were stable. Both eta and epsilon shrinkages were below 20% on all parameters of the final model. The condition number representing model identifiability was 11.23.

### Probability of Target Attainment

**Fig. 4:**
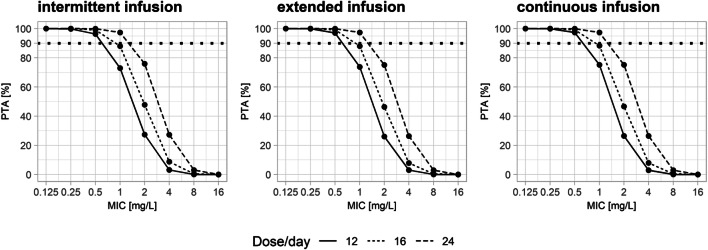
Plots showing probability of target attainment in brain at various doses and MIC levels for different forms of infusion using plasma model from Roberts et al. [10]

MCS indicated that the probability of target attainment was more than 90% for MICs up to 0.5 mg/L for all three simulated doses (12, 16, and 24 g per day). For a MIC of 1 mg/L, PTA was more than 90% only for the doses of 24 g per 24 h. All three types of infusion performed similarly with respect to PTA over the dose range of 12–24 g per day (Fig. 4). These results were not significantly different when other plasma models were used (Fig. 4 and Fig S3 in supplementary materials).

## Discussion

This study quantified the concentrations of piperacillin in the interstitial fluid of brain and generated a PopPK model to describe its pharmacokinetics. MCS suggests that similar CNS exposures are attained in brain ECF with different types of infusion. Furthermore, our results infer that only highly susceptible pathogen can be empirically treated successfully by piperacillin in brain in patients without meningeal inflammation.

The similar piperacillin exposure and related PTA in brain with different types of infusion are an interesting finding (Fig. 4) because many studies based on plasma data have shown superiority of continuous and prolonged infusion over intermittent infusions with regard to PTA in various cohorts of patients including critically ill patients [9, 10]. This result is readily explained by the observation that fluctuations of plasma concentrations are dampened considerably by the transport of the drug to and from the brain, thus mimicking kind of a continuous infusion for the brain. Therefore, continuous infusion would not provide any pharmacokinetic benefit over intermittent infusion to achieve appropriate piperacillin brain concentrations in patients without general meningeal inflammation. This may also apply for other, mainly hydrophilic, anti-bacterials with slow transport into and from the brain and for other tissues with low transfer rates from and to the plasma.

Piperacillin is an anti-pseudomonal penicillin derivative mainly used to treat nosocomial pathogens such as *Pseudomonas aeruginosa*. However, based on a daily dose of 12 g of piperacillin, maximum observed brain concentrations after multiple doses (i.e., about 7.5 mg/L) were far below the concentration expected to be successful in treating *P. aeruginosa* (Table 2). For the same daily dose, MCS showed that the pharmacodynamic target in brain is only achieved for bacteria up to a MIC of 0.5 mg/L, and thus, infections involving only highly susceptible pathogens (Table 2) could be treated with this commonly used dosing regimen. Using higher doses (16–24 g/day) only achieved an acceptable PTA up to a MIC of 1 mg/L. This shows that increasing the dose up to the maximum recommended doses might still not be sufficient to treat CNS infections caused by pathogens with higher MICs (Table 2). Thus, piperacillin might not be an option for prophylaxis for invasive neurosurgical procedures, despite of some previous evidence supporting its use [28]. As an additional problem for its potential use, high variability in piperacillin brain pharmacokinetics was found (Fig S1) which is similar to plasma data where a high variability has also been reported in critically ill patients of various populations [10, 27, 29–31]. Having said this, it is important to mention that brain exposure of piperacillin in meningitis patients is expected to be higher than in hemorrhagic stroke patients because only a small part of the brain is affected as compared to meningitis where the blood–brain barrier is disrupted based on the generalized inflammation [32].

An important consideration for the interpretation of our results is that tazobactam concentrations were not quantifiable. MICs of piperacillin are typically higher than MICs of piperacillin/tazobactam for several pathogens, especially for beta-lactamase producers [33]. In the worst case, tazobactam would not reach the brain and the higher MICs of piperacillin rather than MICs of piperacillin/tazobactam would apply. It is generally believed that piperacillin and tazobactam exhibit similar pharmacokinetics [34] and tazobactam does not affect the pharmacokinetic behavior of piperacillin [35]. However, piperacillin inhibits the cumulative urinary excretion of tazobactam (mediated through OATs transporters) thereby increasing its plasma concentrations [36]. In addition, a recent study reported a high variability in the ratio of piperacillin over tazobactam (ranging from 1 to 10) despite of their high correlation (0.93) [37]. We speculate that this variability might be even higher in brain as compared to the plasma data.

Because microdialysis in vivo recovery experiments could not be carried out due to retrospective nature of the study, brain data were fitted on the basis of in vitro recovery of piperacillin. However, there is some evidence that in vivo recovery might slightly differ from in vitro, usually with lower values in vivo [38]. However, at low flow rates for hydrophilic molecules with relatively low molecular weight like piperacillin, high recovery can be expected. Therefore, the low flow rate used in our study (0.3 µL/min) would explain why our in vitro recovery values are higher as compared to in vivo values (8–40%) reported in the literature [39–41] where flow rates of 1.5–2 µL/min were used. Thus, PTA values estimated in the present study with the assumption of a recovery of 100% are a realistic, albeit conservative estimate. Similarly, we could not estimate brain penetration from area under the concentration time curve (AUC) ratio between brain and plasma (AUC_brain_/AUC_plasma_) of piperacillin due to the lack of plasma data. However, the unavailability of plasma concentrations is not relevant for PTA estimations as also supported by the sensitivity analysis (Fig. S3). Additionally, our simulation results are based on pharmacodynamic targets validated in plasma as brain targets were not available. Small sample size and the presence of BQL values were among the other limitations of our study.

## Conclusion

In conclusion, piperacillin exposure to brain is delayed after initial intravenous infusion, and concentration profiles would be expected to remain similar to different durations of infusion. The results suggest that piperacillin would not be appropriate in most CNS infections, in particular, in pseudomonas CNS infections.

## Electronic supplementary material

Below is the link to the electronic supplementary material.

Supplementary material 1 (DOCX 530 kb)
