# Supplementary material for: Brain Exposure to Piperacillin in Acute Hemorrhagic Stroke Patients Assessed by Cerebral Microdialysis and Population Pharmacokinetics
Source: Neurocrit Care. 2020 Mar 26;33(3):740–8. doi: 10.1007/s12028-020-00947-x (PMC7736006; doi:10.1007/s12028-020-00947-x)
Supplement: Supplementary file 1 — Supplementary material 1 (DOCX 530 kb) [file 12028_2020_947_MOESM1_ESM.docx]

**Supplementary materials**

**Title:** Brain exposure to piperacillin in acute hemorrhagic stroke patients assessed by cerebral microdialysis and population pharmacokinetics

**
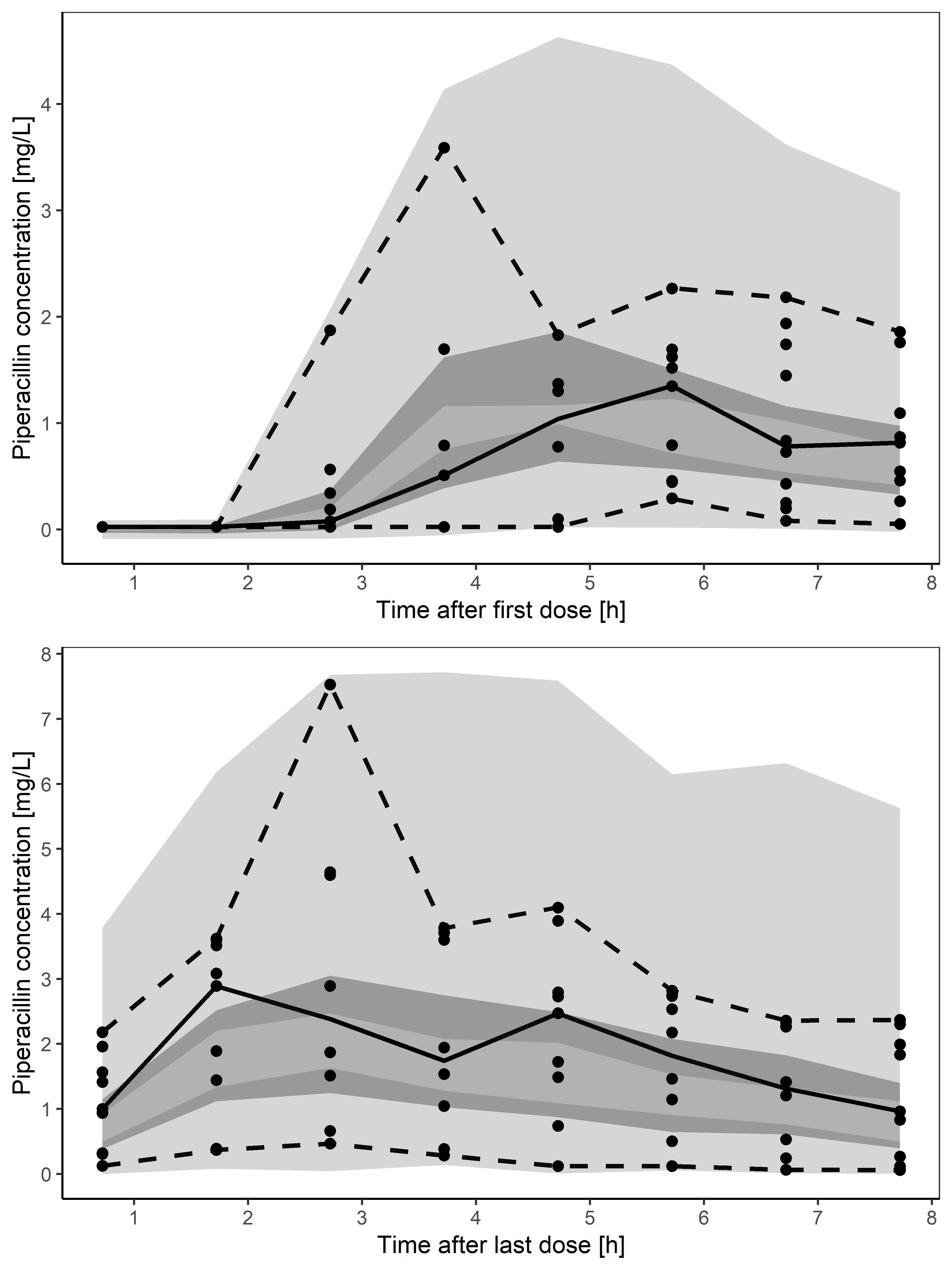
**

**Fig S1.** Visual predictive checks after single dose (upper) and after multiple doses (lower). Black circles mean observed data. Solid line represents the median and upper and lower dashed lines represent the 5^th^ and 95^th^ percentiles of the observed data respectively. While dark and light shaded areas represent predicted median and 95% prediction interval respectively.


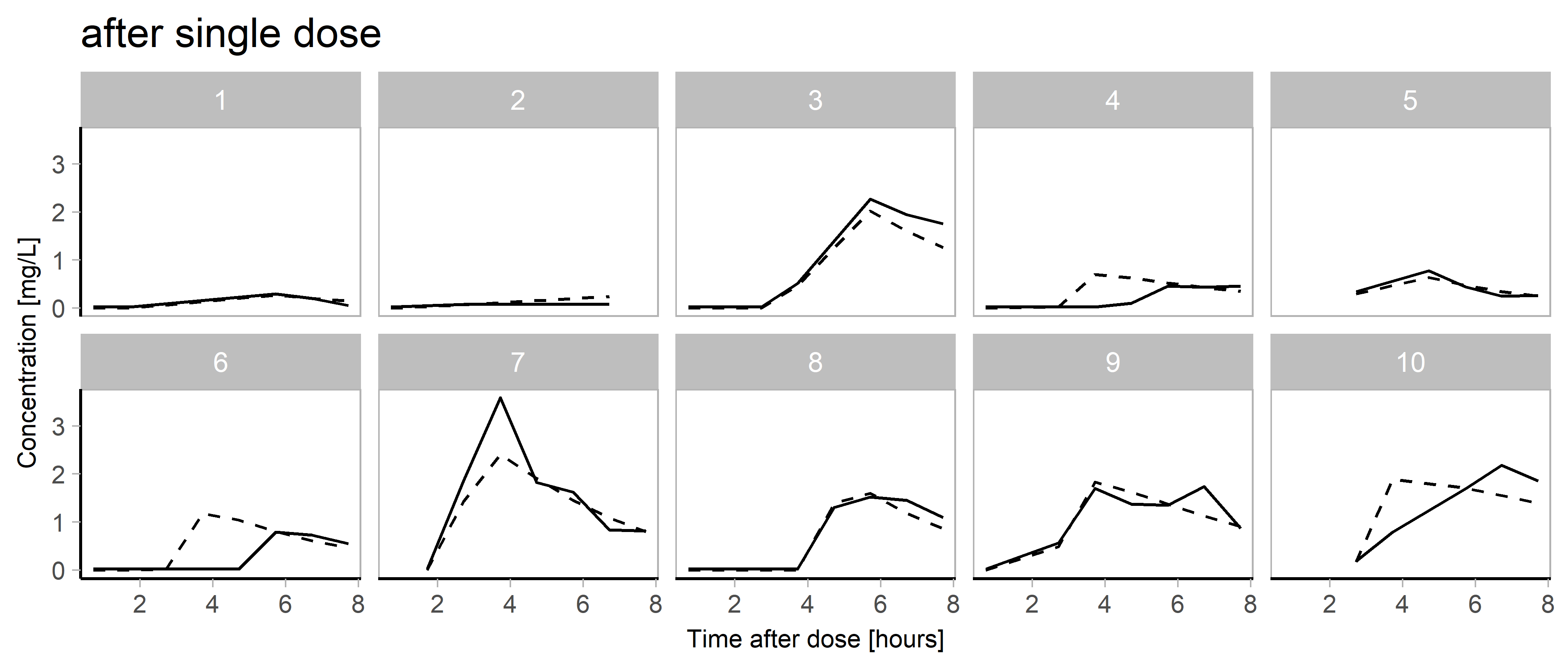

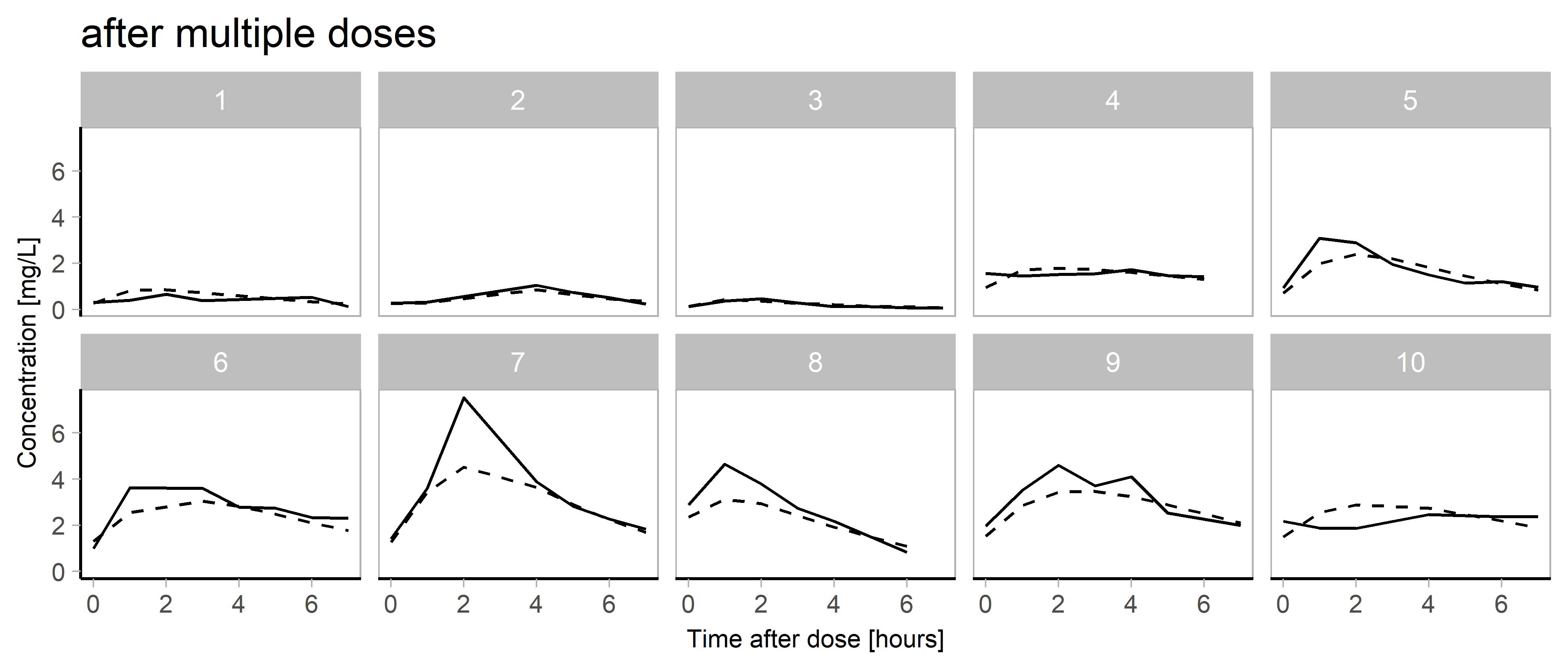


**Fig S2.** Concentration-time plots of individual patients after first dose (upper) and after multiple doses (lower) in brain. Solid line represents observed concentration while dotted line shows predicted concentration.

**
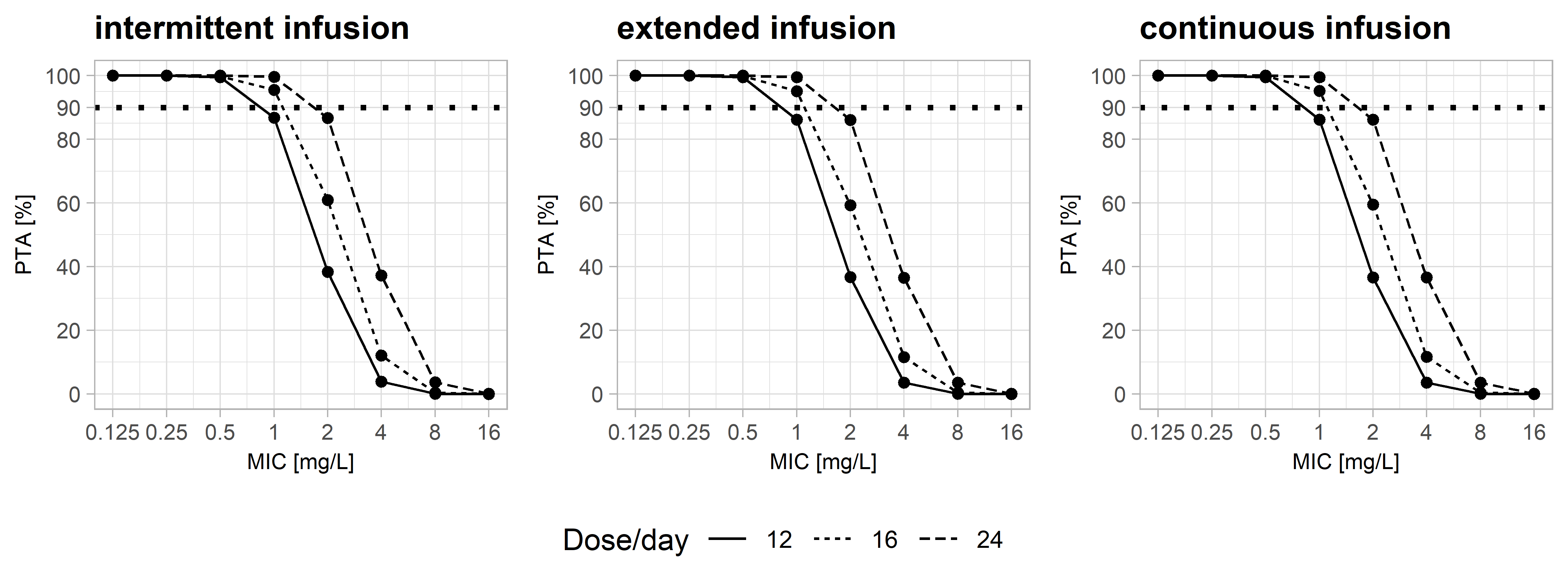

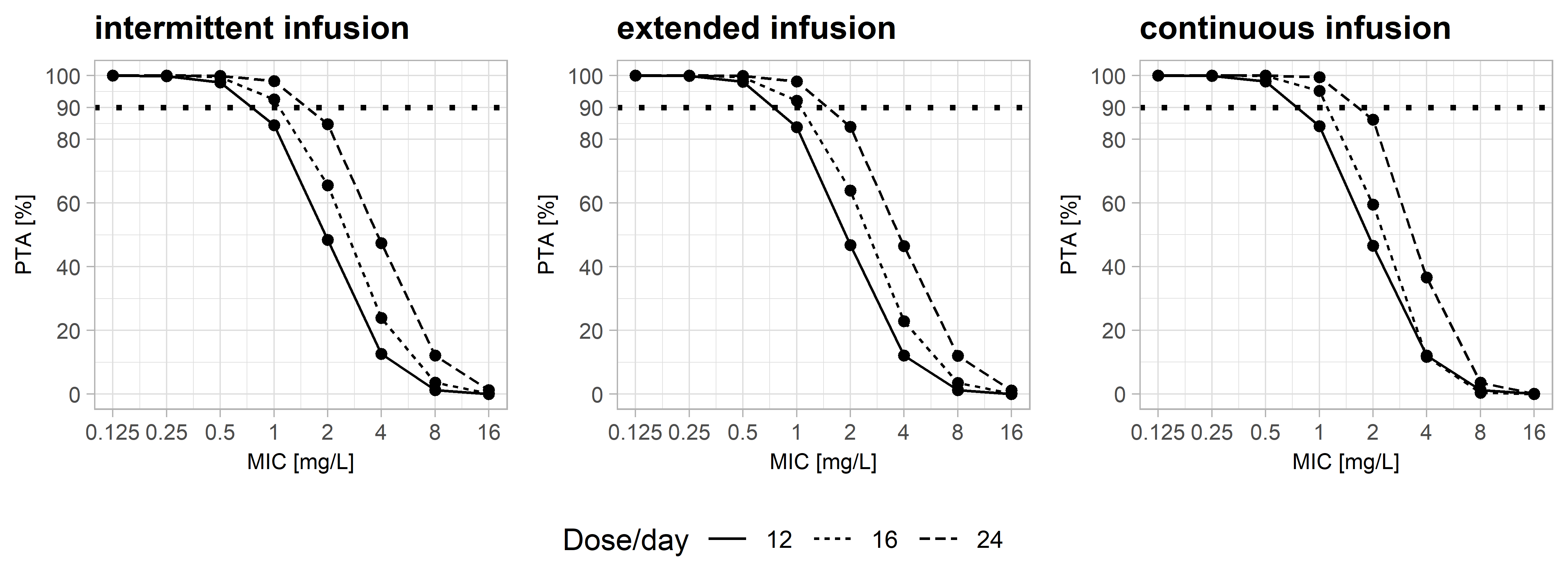
Fig S3.** Results of sensitivity analysis showing that probability of target attainment (PTA) in brain does not depend on the choice of the plasma model. PTA at various doses and MIC levels for different forms of infusion are shown using plasma models from Alobaid et al^1^ (upper) and Öbrink-Hansen et al^2^ (lower).

**References**

1. Alobaid AS, Wallis SC, Jarrett P, et al. Population pharmacokinetics of piperacillin in nonobese, obese, and morbidly obese critically ill patients. Antimicrob Agents Chemother. 2017;61(3).

2. Öbrink-Hansen K, Juul RV, Storgaard M, et al. Population pharmacokinetics of piperacillin in the early phase of septic shock: does standard dosing result in therapeutic plasma concentrations? Antimicrob Agents Chemother. 2015;59:7018–26. Available from: http://www.ncbi.nlm.nih.gov/pubmed/26349823
